# Thrombus Age in Ischemic Stroke With Atrial Fibrillation: Impact on Mechanical Thrombectomy

**DOI:** 10.1161/SVIN.124.001499

**Published:** 2024-12-12

**Authors:** Junpei Koge, Kinta Hatakeyama, Kanta Tanaka, Takeshi Yoshimoto, Masayuki Shiozawa, Yuma Kondo, Kotaro Sakamoto, Naruhiko Kamogawa, Soichiro Abe, Hiroyuki Ishiyama, Tomohide Yoshie, Hirotoshi Imamura, Hiroharu Kataoka, Masafumi Ihara, Toshiyuki Miyata, Kazunori Toyoda, Masatoshi Koga

**Affiliations:** ^1^ Department of Cerebrovascular Medicine National Cerebral and Cardiovascular Center Suita Osaka Japan; ^2^ Department of Pathology National Cerebral and Cardiovascular Center Suita Osaka Japan; ^3^ Division of Stroke Care Unit National Cerebral and Cardiovascular Center Suita Osaka Japan; ^4^ Department of Neurology National Cerebral and Cardiovascular Center Suita Osaka Japan; ^5^ Department of Cardiovascular Medicine Nara Medical University Kashihara Japan; ^6^ Department of Neurosurgery National Cerebral and Cardiovascular Center Suita Osaka Japan

**Nonstandard Abbreviation and Acronym**

MT: mechanical thrombectomy

Mechanical thrombectomy (MT) has allowed the histopathologic analysis of stroke thrombi. Because the biochemical composition of the thrombus changes with time after thrombus formation, the potential influence of thrombus age has attracted attention.^1^ Older thrombi have been reported to be associated with a prolonged time from puncture to reperfusion using MT than fresh thrombi.^1^

In patients with atrial fibrillation (AF), the dominant source of thrombi is believed to be the left atrium. Thrombus age in ischemic stroke with AF may reflect the process of thrombus maturation in the left atrium. Congestive heart failure and higher CHADS_2_ scores are well‐known risk factors for left atrium thrombi in patients with AF.^2^ However, factors affecting thrombus age in patients with AF are unknown. Moreover, the associations between thrombus age in patients with AF and outcomes after MT remain to be elucidated. We aimed to investigate the association of thrombus age with the risk factors for AF‐related ischemic stroke and MT outcomes in ischemic stroke with AF.

The data that support the findings of this study are available from the corresponding author on reasonable request. We retrospectively reviewed consecutive patients with acute ischemic stroke who underwent MT from July 2014 to July 2021 who met the following criteria: (1) patients with known AF or AF diagnosed after stroke onset; (2) patients whose retrieved thrombus specimens were available; and (3) patients who signed a comprehensive consent form for the National Cerebral and Cardiovascular Center Biobank. The requirement for obtaining written informed consent for study registration was waived because of the retrospective design and the use of anonymized data. Ethical approval was obtained from the local institutional review board (R21005).

Retrieved thrombi were immediately fixed in 10% neutral‐buffered formalin and cut into 4‐µm‐thick paraffin‐embedded sections. Serial sections were stained with hematoxylin and eosin. Immunohistochemical staining was performed with primary antibodies against fibrin (mouse monoclonal; Accurate Chemical & Scientific Corp, NY) and glycophorin A (mouse monoclonal, clone JC159; DAKO, CA) for red blood cells, von Willebrand factor (rabbit polyclonal; DAKO) and CD61 (mouse monoclonal; Leica Biosystems) for platelets, and citrullinated histone H3 (rabbit monoclonal, EPR16987; Abcam, Cambridge, UK) for neutrophil extracellular traps. Areas showing immunopositivity for each component were semiautomatically extracted from the digital slides using a color imaging analysis system (WinROOF2001; MITANI CORPORATION, Fukui, Japan), and fractions of the immunopositive area were calculated as the pixel density percentages. Thrombi were classified as fresh or lytic (older) according to the accepted definition^3^: a fresh thrombus (<1 day) consisted of intact granulocytes constituting >80% of the section, whereas a lytic thrombus (≥1 day) was characterized by areas of colliquation necrosis and granulocyte karyorrhexis constituting >20% in the section.

Logistic regression models were created to assess the association of thrombus age with the risk factors and outcomes. For outcomes, the following variables were included on the basis of prior hypotheses: age, sex, baseline National Institutes of Health Stroke Scale score, congestive heart failure, occlusion site, and use of intravenous thrombolysis.

A total of 168 patients (79 women; median age, 79 years) were analyzed. The thrombi were classified as older in 62 patients. The remaining 106 patients had fresh thrombi. Patient characteristics, thrombus components, and outcomes stratified according to thrombus age were presented in the Table. CHADS_2_ score was independently associated with older thrombi, adjusting for sex, dyslipidemia, sustained AF, current smoking, prior antiplatelet use, and prior oral anticoagulant use (adjusted odds ratio [aOR], 1.48 [95% CI, 1.08–2.02]). Older thrombi had higher proportions of fibrin, platelets, von Willebrand factor, and neutrophil extracellular traps than fresh thrombi.[Table svi212985-tbl-0001]

**Table svi212985-tbl-0001:** Patient Characteristics, Thrombus Components, and Outcomes in Relation to Thrombus Age

Variable	Older thrombi (n = 62)	Fresh thrombi (n = 106)	*P* value
Age, y	81 (75–85)	78 (73–83)	0.10
Female sex	32 (52)	47 (44)	0.42
Premorbid mRS score	0 (0–2)	0 (0–1)	0.03
Baseline NIHSS score	19 (13–26)	18 (11–25)	0.41
Admission systolic BP, mm Hg	153 (137–182)	150 (135–171)	0.32
ASPECTS^*^	8 (7–10)	8 (7–10)	0.98
**Medical history**			
Hypertension	52 (84)	79 (75)	0.18
Diabetes	10 (16)	21 (20)	0.68
Dyslipidemia	21 (34)	47 (44)	0.20
Ischemic heart disease	5 (8)	11 (10)	0.79
Congestive heart failure	26 (42)	25 (24)	0.02
Previous stroke/TIA	15 (24)	22 (21)	0.70
CHADS_2_ score	2 (2–3)	2 (1–3)	0.02
CHA_2_DS_2_‐VASc score	4 (3–5)	3 (2–4)	0.007
Sustained AF	36 (61)	56 (53)	0.34
Current smoking^†^	8 (13)	6 (6)	0.18
Prior oral anticoagulants	26 (42)	41 (39)	0.75
Prior antiplatelets	8 (13)	18 (17)	0.52
**Initial site of occlusion**			
Extracranial vessel	4 (4)	7 (11)	0.01
Intracranial ICA	27 (26)	20 (32)	
M1	49 (46)	13 (21)	
M2/M3/A1	20 (19)	16 (26)	
Posterior circulation	6 (6)	6 (10)	
**Laboratory data**			
White blood cell count, /µL	7100 (5000–8450)	6540 (5320–8200)	0.49
Hemoglobin, g/dL	12.8 (11.7–14.2)	12.9 (11.9–14.4)	0.47
Platelet count, ×1000/mm^3^	181 (147–222)	186 (155–216)	0.76
CRP	0.19 (0.09–0.51)	0.17 (0.06–0.52)	0.25
International normalized ratio	1.08 (0.99–1.23)	1.09 (1.00–1.24)	0.80
D‐dimer, µg/mL^†^	2.30 (1.22–5.68)	1.80 (1.00–4.10)	0.11
Brain natriuretic peptide, pg/mL^‡^	248 (162–395)	183 (112–336)	0.03
Left atrial diameter, mm^¶^	42 (39–46)	41 (36–46)	0.27
Ejection fraction, %^§^	60 (55–60)	60 (50‐60)	0.93
Left ventricular end‐diastolic dimension, mm	44 (40–49)	46 (42–49)	0.17
Left ventricular end‐systolic dimension, mm	30 (25–34)	30 (28–35)	0.08
**First‐line MT strategy**			
SR	15 (24)	35 (33)	0.18
CA	25 (40)	35 (33)	
Combined SR and CA	22 (36)	31 (29)	
Manual aspiration through the guide catheter	0 (0)	5 (5)	
Onset to hospital arrival time, min	135 (45–341)	100 (49–323)	0.74
Onset to puncture time, min	214 (115–430)	182 (118–394)	0.67
Intravenous thrombolysis	21 (34)	56 (53)	0.02
**Components of thrombi**			
Erythrocytes, %	40.5 (32.3–47.6)	40.9 (31.3–53.1)	0.69
Fibrin, %	18.8 (13.0–29.2)	14.5 (9.2–22.6)	0.01
Platelets, %	36.3 (21.2–48.6)	27.1 (17.0–40.0)	0.002
von Willebrand factor, %	14.1 (9.0–19.8)	11.4 (7.4–16.9)	0.02
Neutrophil extracellular traps, %^†^	1.41 (0.59–3.35)	0.68 (0.04–2.29)	0.006
**Procedural outcomes**			
First‐pass effect	13 (21)	46 (43)	0.004
Final eTICI ≥2b	53 (86)	101 (95)	0.04
**Clinical outcomes**			
Favorable outcome	25 (40)	77 (73)	<0.001
Unfavorable outcome	17 (27)	12 (11)	0.01

Data are presented as median (interquartile range) or number (percentage).

A1 indicates first segment of the anterior cerebral artery; AF, atrial fibrillation; ASPECTS, Alberta Stroke Program Early CT [Computed Tomography] Score; BP, blood pressure; CA, contact aspiration; CRP, C‐reactive protein; eTICI, extended Thrombolysis in Cerebral Infarction; ICA, internal carotid artery; M1, first segment of the middle cerebral artery; M2, second segment of the middle cerebral artery; M3, third segment of the middle cerebral artery; mRS, modified Rankin Scale; MT, mechanical thrombectomy; NIHSS, National Institutes of Health Stroke Scale; SR, stent retriever; and TIA, transient ischemic attack.

^*^ ASPECTS was obtained using noncontrast CT (n = 50) or diffusion‐weighted magnetic resonance imaging (n = 117).

^†^ One missing value (1 in the fresh thrombi group).

^‡^ Two missing values (2 in the fresh thrombi group).

^¶^ Nine missing values (3 in the older thrombi group and 6 in the fresh thrombi group).

^§^ Five missing values (1 in the older thrombi group and 4 in the fresh thrombi group).

The rates of first‐pass effect (achievement of extended Thrombolysis in Cerebral Infarction score 2c/3 after first pass) (aOR, 0.37 [95% CI, 0.16–0.85]) and final extended Thrombolysis in Cerebral Infarction ≥2b reperfusion (aOR, 0.24 [95% CI, 0.06–0.98]) were significantly lower in patients with older thrombi than in those with fresh thrombi. There were no differences in the rate of any intracranial hemorrhage between the groups. Patients with older thrombi had a lower rate of favorable outcome (modified Rankin scale score of 0–2 or equal to the premorbid modified Rankin scale score at 3 months) (aOR, 0.34 [95% CI, 0.15–0.76]) and a higher rate of unfavorable outcomes (modified Rankin scale score of 5–6 at 3 months) (aOR, 2.94 [95% CI, 1.10–7.83]) than those with fresh thrombi.

In patients showing ischemic stroke with AF, older thrombi were associated with CHADS_2_ score. congestive heart failure, and vascular risk factors, which may play a role in thrombus maturation in the left atrium. Older thrombi were characterized by abundant components associated with thrombectomy resistance, including fibrin, platelets, von Willebrand factor, and neutrophil extracellular traps, which is consistent with a previous study.^1^ A longer time after thrombus formation may cause platelet‐driven clot contraction and fibrin cross‐linking, which increases thrombus stiffness.^4^, ^5^ These factors may be responsible for the substantially lower rates of first‐pass effect and final extended Thrombolysis in Cerebral Infarction ≥2b reperfusion in patients with older thrombi. Moreover, a higher frequency of concomitant congestive heart failure may contribute to poorer functional outcomes in patients with older thrombi. Although there are limitations to this study, including a selection bias, the effect of intravenous thrombolysis on thrombus components, and the small sample size that might have limited the power to detect major differences between the groups, we showed that thrombus age in ischemic stroke with AF varies depending on clinical risk factors. Older thrombi are associated with thrombectomy resistance and poorer functional outcomes.

## Disclosures

Dr Koge received lecture fees from Medtronic and Daiichi‐Sankyo. Dr Yoshimoto received lecture fees from Takeda Pharmaceutical, Nippon Boehringer Ingelheim, Daiichi‐Sankyo, Stryker, and Tonbridge Medical. Dr Yoshie received lecture fees from Medtronic Japan Co, Johnson & Johnson Co, Stryker Japan Co, Terumo Co, and Asahi Intecc Co. Dr Imamura received speakers’ bureau/honoraria from Medtronic Japan Co, Daiichi Sankyo Co, Johnson & Johnson Co, Stryker Japan Co, Terumo Co, and Asahi Intecc Co. Dr Kataoka received research funding from Eizai and lecture fee from Daiichi Sankyo, Otsuka Pharmaceutical, Carl Zeiss, Idorsia Pharmaceuticals, and CSL Behring. Dr Ihara received lecturer fee from Otsuka, and grant support from Panasonic, GE Precision Healthcare, Kyocera Corporation, Towa Pharmaceutical, and Pharma Foods International. Dr Toyoda received lecturer fees from Otsuka, Daiichi Sankyo, Janssen, Bayer, and Bristol‐Myers Squibb. Dr Koga received honoraria from AstraZeneca, Bayer Yakuhin, Daiichi‐Sankyo, Mitsubishi Tanabe Pharma Corporation, and Otsuka Pharmaceutical, and research support from Boston Scientific Corporation, Daiichi‐Sankyo, and Nippon Boehringer Ingelheim, all of which are outside of the submitted work. The other authors report no conflicts.

## Sources of Funding

This study was supported by an Intramural Research Fund of the National Cerebral and Cardiovascular Center (20‐4‐3) and a grant from the JSPS KAKENHI (20K16716 and 24K19537).

## Supporting information


**STROBE Statement**—checklist of items that should be included in reports of observational studies
